# Effect of esomeprazole versus placebo on pulmonary exacerbations in cystic fibrosis

**DOI:** 10.1186/1471-2466-14-21

**Published:** 2014-02-15

**Authors:** Emily DiMango, Patricia Walker, Claire Keating, Maria Berdella, Newell Robinson, Elinor Langfelder-Schwind, Diane Levy, Xinhua Liu

**Affiliations:** 1 Columbia University Medical Center Department of Medicine, 622 West 168th Street, New York, NY 10032, USA; 2 Beth Israel Medical Center Department of Medicine, 10 Nathan D. Perlman Place, New York, NY 10003, USA; 3 Columbia University Mailman School of Public Health, 722 West 168th Street, New York, NY 10032, USA

## Abstract

**Background:**

Gastro esophageal reflux (GER) is common in cystic fibrosis (CF) and may contribute to lung disease. Approximately 50% of patients with cystic fibrosis are being treated with proton pump inhibitors (PPIs).

**Methods:**

In a randomized controlled study in adults, we compared treatment with esomeprazole 40 mg twice daily versus placebo in patients with CF and frequent respiratory exacerbations over a thirty-six week treatment period to determine effect on time to first exacerbation and other health related outcomes.

**Results:**

17 patients without symptoms of GER were randomized and 15 completed the study. 13 subjects underwent 24 hour ambulatory pH probe monitoring; 62% had pH probe evidence of GER. Forty one percent of subjects had a pulmonary exacerbation during the study. There was no significant difference in time to first pulmonary exacerbation (log rank test p = 0.3169). Five of nine subjects in the esomeprazole group compared with 2 of eight subjects in the placebo group experienced exacerbations (esomeprazole vs. placebo: odds ratio = 3.455, 95% CI = (0.337, 54.294), Fisher’s exact test: p = 0.334). There was no change in Forced Expiratory Volume in one second, Gastroesophageal Symptom Assessment Score or CF Quality of Life score between the two treatment groups.

**Conclusions:**

There was a trend to earlier exacerbation and more frequent exacerbations in subjects randomized to esomeprazole compared with placebo. The effect of proton pump inhibitors on pulmonary exacerbations in CF warrants further investigation.

**Clinical trials registration:**

Clinicaltrials.gov, NCT01983774

## Background

Gastroesophageal reflux (GER), both symptomatic and silent, is frequent in patients with cystic fibrosis (CF), and is often regarded as playing a role in the pathogenesis of CF related lung disease [1-4]. The overall prevalence of GER in CF is not well established, but is reported to be as high as 80% when diagnosed by esophageal pH-probe monitor in CF adults [3,5]. One study reported that 91% of patients with CF awaiting lung transplant had evidence of GER by pH probe monitoring [6]. Symptoms of lung disease in CF may overlap with pulmonary symptoms of gastroesophageal reflux, making it difficult to distinguish between the two conditions and often leading to treatment of both conditions. In 2010 in the US, 48% of adults and 51% of children with CF were being treated with proton pump inhibitors [7].

Several studies have suggested that patients with CF who have GER have more severe lung disease with lower pulmonary function and increased numbers of respiratory exacerbations [2,8]. In a prospective study, Button etal demonstrated that children with CF receiving modified chest physiotherapy with avoidance of head in the tilt down position not only had reduced episodes of GER as measured by ambulatory pH probe, but also had reduced need for antibiotics, reduced number of hospital days and improved lung function over a five year period [9]. The European Epidemiologic CF Registry reported that patients with CF and GER had lower pulmonary function than those without GER [8]. A recently conducted retrospective study of Nissen fundoplication in patients with CF and GER showed a significant decline in pulmonary exacerbations and improvement in forced expiratory volume in one second (FEV_1_) during the two years following surgery compared to the two years preceding surgery [10]. Despite considerable evidence that GER is common in CF and may be associated with more severe lung disease, the effect of acid suppressor therapy on improving lung function and reducing pulmonary exacerbations has not been prospectively studied.

Proton pump inhibitors (PPIs) suppress the production of gastric acid and several studies have tested their effectiveness in improving pulmonary outcomes in chronic respiratory diseases. Studies of PPI therapy in asthma have inconsistently demonstrated beneficial effects [11,12], and retrospective studies in idiopathic pulmonary fibrosis suggest stabilization of lung function and improved survival with acid suppression [13,14] , Among individuals with CF , PPIs are likely initiated for a variety of reasons including improved efficacy of pancreatic enzymes in a higher pH environment, as well as treatment of cough or other respiratory or gastrointestinal complaints thought to be possibly caused by GER. Use of these agents however, may be associated with risk [15,16]. Use of PPIs in both hospitalized and ambulatory patients has been shown to be associated with an increased risk of pneumonia [15-18]. Furthermore, PPIs have been implicated in accelerated bone loss [19,20]. We compared treatment with esomeprazole versus placebo in a pilot study of patients with CF and frequent respiratory exacerbations to determine whether suppression of gastric acid leads to longer time to first pulmonary exacerbation and improvements in other health related outcomes.

## Methods

We conducted a randomized, placebo-controlled double blind trial of esomeprazole in adult patients with cystic fibrosis. Adults with cystic fibrosis were enrolled from the clinical practices of two adult cystic fibrosis programs in New York City. Inclusion criteria were age of 18 years or older and two to four respiratory exacerbations per year requiring oral and/or intravenous antibiotics for each of the two years prior to study entry. At the time of enrollment, subjects had to have been on a stable maintenance medical regimen for at least six weeks. Participants were excluded if they were being treated with PPIs, were receiving enteral feeds, had smoked cigarettes within the previous six months, had previous anti-reflux surgery or clinical indications for acid-suppressor treatment (i.e. two or more episodes per week of heartburn requiring antacids). Participants were also excluded if they were being treated with medications that interact with proton pump inhibitors (azoles, iron, anti-coagulants), were pregnant or had a pulmonary exacerbation requiring antibiotics within the previous two weeks. All participants provided written informed consent statements that had been approved by the Columbia University Institutional Review Board (IRB AAAC8262) and the Beth Israel Medical Center Institutional Review Board (IRB 074-10).

After the screening visit, those subjects who met eligibility criteria were enrolled in a 2 week run-in period during which time they underwent 24-hour ambulatory pH probe monitoring. Calibrated pH probes were placed in the distal esophagus using esophageal manometry, 5 cm above the lower esophageal sphincter. Criteria for an acceptable study included total recording time of at least 16 hours, with at least one meal and 2 hours of recumbency. A study was considered positive for distal GER if the distal pH was less than 4 more than 5.8% total time, or more than 8.2% of upright time, or more than 3.5% of supine time [21,22]. Meal times were excluded in the analysis to avoid false-positive data. A single gastroenterologist at each of the two centers reviewed studies; study subjects and study investigators were blinded to the results.

Fourteen days after screening, subjects were randomly assigned in a 1:1 ratio to receive either esomeprazole 40 mg twice daily or matching placebo, regardless of pH probe results. The Columbia University Research Pharmacy prepared study medication. At the randomization visit, baseline spirometry, CF related quality of Life (CFQ-R QOL) [23] and Gastroesophageal Symptom Assessment Score (GSAS) measuring number and severity of reflux symptoms [24] were collected. Randomization was stratified based on study center and FEV_1_ decile. Primary outcome measure was time to first pulmonary exacerbation. Secondary outcomes included exacerbation rate, change in FEV_1_, forced vital capacity (FVC), CFQ-R QOL score and GSAS score. After randomization, participants returned to the clinic every six weeks for 36 weeks. Outcome measures were re-assessed at 12, 24 and 36 weeks after randomization. Subjects were instructed to notify the study site if they had signs or symptoms of a pulmonary exacerbation or if they were treated for a pulmonary exacerbation. Pulmonary exacerbation was defined as initiation of treatment with intravenous or oral antibiotics for 7 or more days based on respiratory symptoms at the discretion of the treating physician [24,25]. Additionally, at each visit, subjects answered questions from a checklist to ensure that all exacerbation events were captured.

## Statistical analysis

Summary statistics were calculated for sample characteristics of each treatment group. Fisher’s exact method and Wilcoxon rank sum test was used to detect treatment group differences in baseline binary and quantitative variables respectively. Kaplan-Meier product limit method was used to estimate cumulative probability curve for time to first exacerbation in each treatment group and log rank test was used to detect group difference in the curve. Rate of exacerbation defined as number of exacerbations per person year was calculated by treatment group and negative binomial model was used to examine treatment group differences. Linear model with repeated measures were used to examine treatment group difference in FEV_1_, FVC, CFQ-R and GSAS over time. For participants who were withdrawn after randomization, longitudinal analyses compared each value at the start of the treatment period to the last observed value carried forward for each variable examined.

## Results

**Figure 1:**
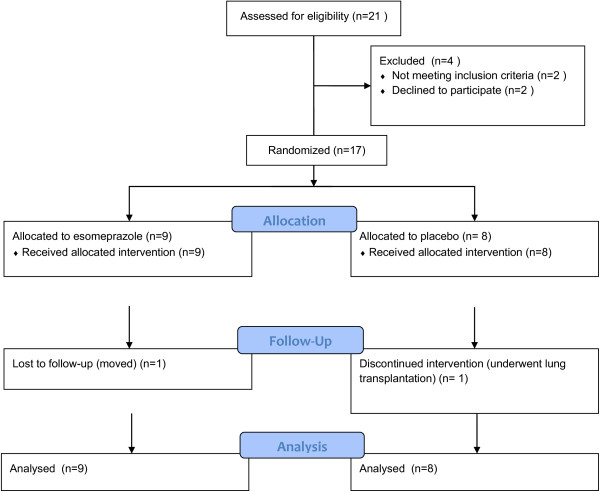
Flow diagram for screened and enrolled subjects.

Twenty one subjects were screened; two subjects withdrew consent before randomization, one subject was ineligible based on daily symptoms of GER (an indication for acid suppressor therapy) and one subject was ineligible due to frequency of exacerbations being above the threshold for enrollment. Of the 17 subjects who were randomized, four were unable to tolerate insertion of the pH probe but remained in the study. Fifteen subjects completed the study; all randomized subjects are included in the analysis (Figure 1). There were no significant differences between subjects randomized to placebo and those randomized to esomeprazole, though the placebo group tended toward lower lung function, more frequent exacerbations and lower body mass index (BMI) (Table 1). Of the subjects who underwent 24 hour pH probe monitoring, five of eight subjects (62.5%) in the esomeprazole group and three of five subjects (60%) in the placebo group had probe evidence of GER. There were no significant differences in baseline characteristics between subjects with and without evidence of distal GER (Table 2).

**Table 1:** Baseline characteristics of subjects by treatment assignment

	**Esomeprazole (n = 9)**	**Placebo (n = 8)**	**p value**
Reflux present on pH probe	5/8 (62%)	3/5 (60%)	0.42
Male (%)	67	75	0.38
Pseudomonas present (%)	89	62	
MRSA present(%)	0	25	
	Mean + SD	Mean + SD	
Age (years)	35.72 + 9.6	32.81 + 5.84	0.41
BMI	24.25 + 4.72	21.84 + 3.02	0.21
# exacerbations past 2 years	4 + 0 (0)	5.5 + 1.4 (SD)	
FEV1 (%)	58 + 19	46 + 21	0.14
FVC (%)	74 + 20	71 + 16	0.88
FEV1/FVC	0.63 + 0.10	0.56 + 0.15	0.26
GSAS distress score	0.99 + 0.61	0.88 + 1.03	0.28
CFR-QOL score	72.28 + 10.32	77.85 + 18.86	0.34

**Table 2:** Comparison of subjects with and without gastroesophageal reflux as measured by 24 hour ambulatory pH probe

	**+pH probe (n = 8)**	**-pH probe (n = 5)**	**p value**
Age	33.8 (4.37)	37 (16.5)	0.59
FEV1 (%)	51 (17)	59 (20)	0.45
BMI	23.5 (2.7)	21.8 (5/2)	0.43
GSAS	0.65 (0.29)	0.59 (0.21)	0.88
+ exacerbations previous two years	5.5 (1.4)	4 (0)	0.33

**Figure 2:**
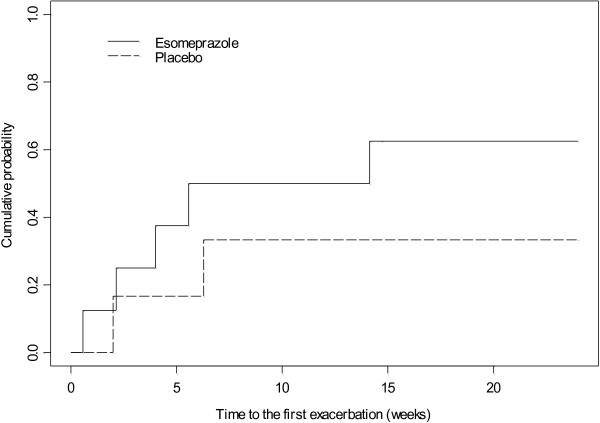
Time to first exacerbation in treatment group assigned to esomeprazole versus placebo. Log rank test p = 0.3169.

**Figure 3:**
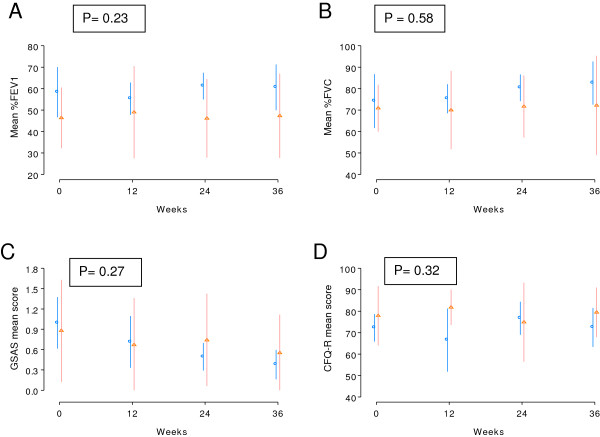
**A. Forced Expiratory Volume in 1 second (FEV1) over treatment period. B.** Forced Vital Capacity (FVC) over treatment period. **C.** Gastroesophageal Symptom Assessment Score (GSAS) over treatment period. **D.** Cystic Fibrosis Quality of Life – revised (CFQ-R) score over treatment period. **Blue lines:** esomeprazole group; mean with standard deviation. **Red lines:** placebo group; mean with standard deviation.

Forty one percent of 17 subjects had a pulmonary exacerbation during the study. Five of nine subjects in the esomeprazole group compared with 2 of 8 subjects in the placebo group experienced exacerbations (esomeprazole vs. placebo: odds ratio = 3.455, 95% CI = (0.337, 54.294). There was no significant difference in time to first pulmonary exacerbation between the esomeprazole and placebo groups (log rank test p = 0.3169) (Figure 2). Similarly, there was no significant difference between groups in exacerbation rate during the study period (2.04 exacerbations per person year in esomeprazole group 95% CI (1.33, 4.14) compared with 0.59 exacerbations per person year in placebo group (95% CI (0.19, 1.82), p = 0.07. There was no significant change in FEV_1_ percent predicted or FVC percent predicted in either group over the study period, p = 0.23 and 0.58, respectively, and there was no difference between groups in change in FEV_1_ or FVC percent predicted from baseline to end of study (Figure 3). GSAS and CFQ-R score did not change significantly over the study period (p = 0.27 and 0.32, respectively) and there was no difference in change in scores between the two treatment groups.

## Discussion

Individuals with CF have many predisposing factors to the development of GER including airway hyperinflation, frequent cough, hyperalimentation, delayed gastric emptying, high fat diet and positional changes related to performance of chest physiotherapy [25]. Twenty-four hour esophageal pH monitoring has the highest sensitivity and specificity for the detection of GER and is widely regarded as the gold standard for quantifying esophageal pH. We demonstrate that the majority of patients with cystic fibrosis in our cohort have evidence of distal esophageal reflux as measured by esophageal pH monitoring despite absence of symptoms. In the small prospective study reported here, suppression of gastric acid with esomeprazole did not lead to significant improvement in pulmonary outcomes. An unanticipated finding of this study was a trend to earlier exacerbation and more frequent exacerbations in patients randomized to esomeprazole compared with placebo.

In 2010, the Cystic Fibrosis Foundation Patient Registry reported that 50.7% of children less < 18 years and 48.2% of adults > 18 years were being treated with proton pump inhibitors. Though studies have suggested that treatment of GER is associated with improvement in other lung diseases, prospective studies have not been conducted in CF to determine whether reducing gastric pH has a beneficial effect on pulmonary exacerbations or other health related outcomes. The possible mechanisms whereby gastroesophageal reflux leads to respiratory symptoms in CF and other chronic lung diseases have not been established. Some investigators speculate that reflux into the esophagus, particularly in the supine position, results in intermittent aspiration of acidic stomach contents into the airways compounding the effects of the vicious cycle of inflammation, infection and progression of lung disease that has been well described in CF. Mendez, et al demonstrated that even after lung transplantation, 90% of patients with CF had evidence of GER compared with only 54% of patients who underwent lung transplant for other diseases. The majority of CF patients had evidence of proximal and distal GER [26]. Tracheal acidification has in fact been demonstrated in adults with CF while in the supine position [27]. It is further hypothesized that afferent receptors within the esophageal mucosa, when stimulated by exposure to acid, trigger outputs along motor neurons to the respiratory muscles and tracheobronchial tree, resulting in cough, bronchospasm and perhaps even increase in neutrophilic airway inflammation [28,29]. A relationship between GER and the development of obliterative bronchiolitis after lung transplantation, with improved allograft function after Nissen fundoplication has been reported by Davis and colleagues [30]. However, a large prospective study of the effect of PPIs on asthma exacerbations did not show an improvement in asthma outcomes [11].

PPIs address only the acid component of reflux, and there is evidence that non-acid reflux, such as bile salts from the small intestine, may also be lung irritants. Tamhankar and others have demonstrated that omeprazole does not reduce the number of reflux episodes or their duration, but acts to convert acid reflux to less acid reflux [31]. Doumit et al showed that among children with CF, 63% of reflux episodes were acid compared with 37% which were non acid [32]. In a study by Pauwels, et al, 56% of patients with CF had bile acids in the sputum, providing evidence for the aspiration of duodenogastric contents [25]. Furthermore, concentration of bile acids correlated with neutrophil elastase in sputum, degree of lung function impairment and need for IV antibiotic treatment.

PPIs have the potential to increase the incidence of hospital and community acquired pneumonia, as demonstrated by several retrospective studies of PPI use in both the in-patient and outpatient setting [15,16]. Individuals with CF have chronic airway infections with a host of pathogens, notably *Pseudomonas aeruginosa* and *Staphylococcus aureus*. Despite widespread use of PPIs in this patient population, their safety and effect on pulmonary outcomes have not been studied.

Our randomized placebo controlled double blind study of the effect of proton pump inhibitors on pulmonary exacerbations in a group of patients with CF and a known history of recurrent exacerbations was designed as a feasibility study and was underpowered to demonstrate a significant effect on respiratory outcomes. We demonstrated that in a population of patients with CF and recurrent pulmonary exacerbations, 60% of patients have asymptomatic acid GER. These results are consistent with those reported by Brodzicki et al where 55% of children with CF had GER, despite the absence of symptoms in many of those patients [33]. There was a trend toward shorter time to first pulmonary exacerbation and higher exacerbation rate in patients randomized to esomeprazole compared with placebo, despite that fact that the placebo group had more frequent exacerbations during the two years prior to study enrollment . Though the study enrolled only subjects with frequent pulmonary exacerbations (between 2 and 4 per year), there was a relatively low incidence of pulmonary exacerbations during the treatment period in that only 42% of subjects experienced an exacerbation over a thirty-six week period. This may be related to the introduction of new therapies during the study period, such as hypertonic saline and inhaled aztreonam lysine. [34,35].

Our study hypothesized that gastric acid suppression would prolong time to first pulmonary exacerbation, thus adequate gastric acid suppression was an essential component of the study design. Esomeprazole was selected because of its high potency for gastric acid suppression; the twice-daily dose of 40 mg has been shown to effectively suppress gastric acid in about 95% of patients [30]. 36 weeks was chosen for study duration to allow long enough follow up time for development of respiratory exacerbation in the majority of patients. Our study findings are limited by small sample size without adequate power to detect significant differences between subjects treated with esomeprazole compared with placebo. However, trends regarding frequency of exacerbation and time to exacerbation were consistent in the esomeprazole group. The fact that our results align with reports from several retrospective studies demonstrating an increased risk of lower respiratory tract infections in patients taking PPIs, and that patients with cystic fibrosis chronically harbor bacterial pathogens and develop recurrent pulmonary exacerbations, suggests that further investigation into the possible effects of PPIs on pulmonary infections in CF is warranted.

This work was previously presented in Abstract form at the North American Cystic Fibrosis Conference 2012 [33].

## Conclusion

Asymptomatic gastroesophageal reflux is present in the majority of patients with cystic fibrosis. Risk and benefits of acid suppressive agents in cystic fibrosis require further study.

## Competing interests

Emily DiMango serves on the Advisory Board of Gilead Pharmaceuticals. Patricia Walker serves as a consultant to Gilead Pharmaceuticals. Claire Keating reports no Conflict of Interest. Maria Berdella reports no Conflict of Interest. Bryce Robinson reports no Conflict of Interest. Elinor Langfelder-Schwind reports no Conflict of Interest. Diane Levy reports no Conflict of Interest. Xinhua Liu reports no Conflict of Interest.

## Authors’ contributions

ED and PW developed the study protocol and oversaw all aspects of the study. CK, MB, ELS and NR conducted study visits and assisted with data analysis. DL and XL performed statistical analysis for the study. All authors read and approved the final manuscript.

## Pre-publication history

The pre-publication history for this paper can be accessed here:

http://www.biomedcentral.com/1471-2466/14/21/prepub
